# Factor VIII inhibitors in hemophilia A treated with emicizumab: longitudinal follow-up of outcomes

**DOI:** 10.1016/j.rpth.2023.100278

**Published:** 2023-06-14

**Authors:** Sarina Levy-Mendelovich, Nitzan Atia, Ivan Budnik, Assaf Arie Barg, Einat Avishai, Omri Cohen, Tami Brutman-Barazani, Tami Livnat, Gili Kenet

**Affiliations:** 1 National Hemophilia Center and Coagulation Unit, Sheba Medical Center, Tel Hashomer, Israel; 2 Amalia Biron Research Institute of Thrombosis and Hemostasis, Sheba Medical Center, Tel Hashomer and Sackler Faculty of Medicine, Tel Aviv University, Tel Aviv, Israel; 3 The Sheba Talpiot Medical Leadership Program, Sheba Medical Center, Tel Hashomer, Israel; 4 Department of Internal Medicine, Division of Hematology/Oncology, University of Iowa, Iowa City, Iowa, USA

**Keywords:** emicizumab, FVIII, FVIII inhibitor, hemophilia A, immune tolerance induction

## Abstract

**Background:**

Using emicizumab in lieu of immune tolerance induction (ITI) for patients with hemophilia A (HA) and factor (F)VIII inhibitors has been well described. However, decisions regarding ITI initiation, regimen, and preservation of tolerance remain to be elucidated.

**Objectives:**

To study the course of FVIII inhibitors in patients with HA and a history of FVIII inhibitors receiving emicizumab prophylaxis.

**Methods:**

Patients with HA, with and without FVIII inhibitors, initiating emicizumab prophylaxis were prospectively followed up in our center. All patients with current or previous inhibitors were included in this analysis. Plasma samples for FVIII inhibitor assays were obtained every 3 to 6 months or following FVIII exposure. Patients documented annual bleeding rate and any FVIII exposure days (EDs).

**Results:**

Of 162 emicizumab-treated participants, 51 met the inclusion criteria. A decrease in annual bleeding rate was observed in all 51 participants followed up for a median of 3.3 years, with 31 breakthrough bleeding episodes reported in 22 of 51 participants. FVIII inhibitor level transiently increased following FVIII exposures in 5 of 15 failed ITI participants. Eight of 21 participants who did not undergo ITI were exposed to FVIII (1-2 EDs)), and 1 of these 8 participants demonstrated increased FVIII inhibitor levels after head trauma (following 1 ED). Among participants who underwent successful ITI, 8 of 15 patients were exposed to FVIII over a total of 13 EDs (1-2 ED(s) each) for traumatic breakthrough bleeds. In all these participants, inhibitor levels remained zero, indicating successful tolerance maintenance.

**Conclusion:**

Our longitudinal follow-up of emicizumab-treated patients with HA and FVIII inhibitors shows that occasional exposure to FVIII may induce a transient anamnestic response. Nonetheless, no FVIII inhibitor recurrence was noted following FVIII exposures in patients who underwent successful ITI.

## Essentials

• Data regarding maintenance of immune tolerance in patients treated with emicizumab are scarce.
• Patients with hemophilia A and FVIII inhibitors who initiated emicizumab were followed up for 3 years.
• No inhibitor recurrence was noted among fully tolerized patients.
• Successfully tolerized patients with hemophilia A treated with emicizumab did not lose tolerance.

## 1 Introduction

Hemophilia A (HA) is a genetic, X-linked, severe bleeding disorder characterized by spontaneous or traumatic bleeding due to coagulation factor (F)VIII deficiency. Patients may experience recurrent hemarthrosis, leading to severe joint arthropathy at a young age [1]. Repeated intravenous coagulation factor concentrate (CFC) infusions had been the backbone of prophylaxis for patients with severe HA (SHA), in order to avoid the vicious cycle of bleeding and inflammation, leading to progressive joint damage [[2], [3], [4]]. Notably, intravenous access may often be challenging in young children [5]. Furthermore, despite CFC prophylaxis, patients with HA still experience breakthrough and traumatic bleeding episodes [6]. Finally, approximately 30% of patients with SHA develop anti-FVIII antibodies (inhibitors) that may render CFC treatment futile. Immune tolerance induction (ITI) is a treatment strategy that aims to eliminate inhibitors to FVIII in these patients. However, ITI yields eradication of FVIII inhibitors in only about two-thirds of patients [6,7] and requires good venous access for frequent FVIII administration, involving high cost and central venous line–associated risks. Moreover, maintenance of tolerance through FVIII prophylaxis after ITI may be required to prevent the recurrence of inhibitors [8].

The concept of non replacement therapy was recently introduced into hemophilia care, and several clinical trials are aimed at restoring hemostasis by rebalancing the coagulation factors and natural inhibitors [9]. Emicizumab (Hemlibra, Roche) is a humanized immunoglobulin G4 bispecific antibody with affinity to FIX/FIXa and FX. It mimics the cofactor activity of FVIII by bridging the 2 factors [[9], [10], [11], [12]]. The HAVEN phase III clinical studies, conducted in both adult and pediatric patients with HA, have established the safety and efficacy of emicizumab [13,14]. Thus, following the Food and Drug Administration and European Medicines Agency approval, emicizumab prophylaxis is currently becoming the standard of care for patients with HA, with and without inhibitors [15]. Currently, inhibitor eradication remains the recommended approach among hemophilia treaters, with clinical and economic justification for using emicizumab to prevent bleeding during ITI. This position was recently endorsed by the UK Haemophilia Doctors’ Organization [8]. Nonetheless, following emicizumab prophylaxis initiation, the majority of patients and their caregivers may choose to refrain from continued FVIII therapy.

Our hemophilia treatment center longitudinally follows a large cohort of patients with SHA, with and without FVIII inhibitors, who are prophylactically treated with emicizumab. In our center, the majority of children with HA and inhibitors, who are offered emicizumab as an alternative to ITI, prefer this option. The question of tolerance preservation in patients on emicizumab prophylaxis when occasionally re-exposed to FVIII on demand due to breakthrough bleeding episodes, surgery, or trauma deserves further evaluation.

Furthermore, there is limited data regarding the impact of emicizumab prophylaxis on patients with HA who have undergone successful ITI and patients with HA who have not undergone ITI or have failed ITI. The aim of the current analysis was to examine the course of FVIII inhibitors in patients treated with emicizumab prophylaxis and FVIII on demand.

## 2 Methods

### 2.1 Patients

The Israeli National Hemophilia Center treats approximately 700 patients, including 600 patients with HA. One hundred sixty-two patients with SHA, with and without FVIII inhibitors, initiating emicizumab prophylaxis were prospectively followed up in our center. All patients with current or previous inhibitors were included in this analysis. All patients with HA, aged 1 month to 80 years, with either FVIII inhibitors or a history of FVIII inhibitors were eligible for the current study, regardless of whether or not they underwent ITI. Of note, inhibitor was defined as “positive” following at least 2 consecutive measurements over 0.5 BU. ITI was applied according to a low-dose regimen (50 units/kg thrice a week) [8]. Success or failure of ITI was defined according to consensus recommendations as previously published [16], after 1 to 3 years of treatment. Partially tolerized patients were included in the failed ITI group.

All patients in the successful ITI group were on regular FVIII prophylaxis with 50 IU/kg thrice weekly prior to emicizumab initiation.

Emicizumab therapy was initiated and maintained per standard protocol as previously described [17]. The patients were instructed to contact and consult the hemophilia center about any physical trauma, bleeding, or other adverse events. The study team conducted weekly telephone calls to each patient/family in order to follow therapy outcomes. To compensate for variable times within the study follow-up, the annual bleeding rate was calculated for each patient.

Bleeding episodes or surgical interventions were treated with additional FVIII (30-50 IU/kg) or recombinant FVIIa (90-120 mcg/kg) doses.

The study was approved by the institutional review board of Sheba Medical Center in accordance with the Declaration of Helsinki (protocol code 5858-19-SMC), and the participants or participants’ legal guardians provided informed consent.

### 2.2 Laboratory methods

Participants’ plasma samples were collected prior to initiation of emicizumab therapy and followed up every 3 months. Samples were analyzed for activated partial thromboplastin time, FVIII activity, and FVIII inhibitors using chromogenic FVIII activity assay [18]. Emicizumab concentration in participants’ plasma was measured by a modified version of the traditional activated partial thromboplastin time–based FVIII 1-stage clotting assay calibrated against emicizumab, which includes a dedicated plasma emicizumab calibrator as well as a plasma-based control [18]. Inhibitor tests were repeated after every FVIII exposure day (ED).

### 2.3 Statistical analysis

Statistical analysis was performed using IBM SPSS Statistics (version 23.0; IBM Corp). Continuous variables are presented as median (IQR or range, as indicated). Categorical variables are presented as counts and/or ratios. The Mann–Whitney *U* test and Kruskal–Wallis test followed by Dunn’s post hoc test were used to compare 2 and 3 independent patient groups, respectively, for continuous variables, and the resulting *P* values were adjusted for the number of time points by Sidak’s correction. The Friedmann test followed by Dunn’s post hoc test was used to compare 1 patient group for continuous variables across 3 or more time points of the study. Two-tailed *P* values <.05 were considered statistically significant.

## 3 Results

Of 162 patients with HA treated with emicizumab prophylaxis and longitudinally followed up at our hemophilia center, a total of 51 met the inclusion criteria and were included in our study cohort. All participants continue follow-up at our center to date. In all participants, FVIII inhibitor was diagnosed during childhood, prior to 2019. All patients diagnosed with confirmed high responding inhibitor (>5BU) were offered ITI. Among patients that underwent ITI it was initiated no later than 1 year after the diagnosis. This cohort was stratified into 3 subgroups as follows: participants with FVIII inhibitor who started emicizumab after failing ITI (partially tolerized participants were considered as failed ITI), participants with a history of FVIII inhibitor following successful previous ITI, and patients with newly diagnosed FVIII inhibitors in whom ITI was deferred (as per a shared decision process between the physician and parents) and eventually refused by the parents, thus referred to as “No ITI”. Notably, these participants switched to emicizumab prophylaxis immediately after inhibitor detection and were never treated with FVIII prophylaxis.

Three patients were considered partially tolerized; of these, 2 had inhibitor levels ranging between 0.5 and 1 BU and 1 had increased clearance of FVIII.

The demographic data of all participants are shown in Table 1. Participants deferring ITI were significantly younger at the time of emicizumab prophylaxis initiation than those who previously underwent successful ITI (*P* = .04). Median historical inhibitor peak levels were similar (*P* > .10) among participants with either previous successful or failed ITI (25 and 32.5 BU, respectively) and participants who refused ITI (16 BU).

**Table 1:** Demographic and clinical characteristics of our study cohort.

Demographic and clinical criteria	Failed ITI^a^ *N* = 15	Successful ITI^b^ *N* = 15	No ITI *N* = 21	*P* value
Race (Caucasians)	*n* = 15	*n* = 15	*n* = 21	
Age of initiation of emicizumab, (y), median (IQR)	5.5 (1.1; 31.4)	14.6 (9.5; 29.4)	2 (0.8; 19.8)	0.045
Inhibitor peak level, BU, median (range)^c^	21.5 (0.5-900)	25 (0.5-284)	16 (0.5-192)	>0.99
Inhibitor level before emicizumab, BU median (IQR)^d^	4.0 (1.1-12.0)	0.0 (0.0-0.5)	1.3 (0.5-5.1)	<0.001
Time from stop or completion of ITI till emicizumab initiation, median (IQR) (y)	6 (2-10)	13 (9-27)		
ABR prior to emicizumab, median (IQR)	7 (3-10)	1 (0-7.75)	3 (1-6)	0.036

ABR, annual bleeding rate; ITI, immune tolerance induction.

a Failure of ITI was defined as no full success after 3 years of treatment.

b The median time to successful ITI was 1 year (IQR, 9-15 months; range, 5 months-2.5 years).

c Historical peak inhibitor level (BU).

d Kruskal–Wallis test.

**Figure 1:**
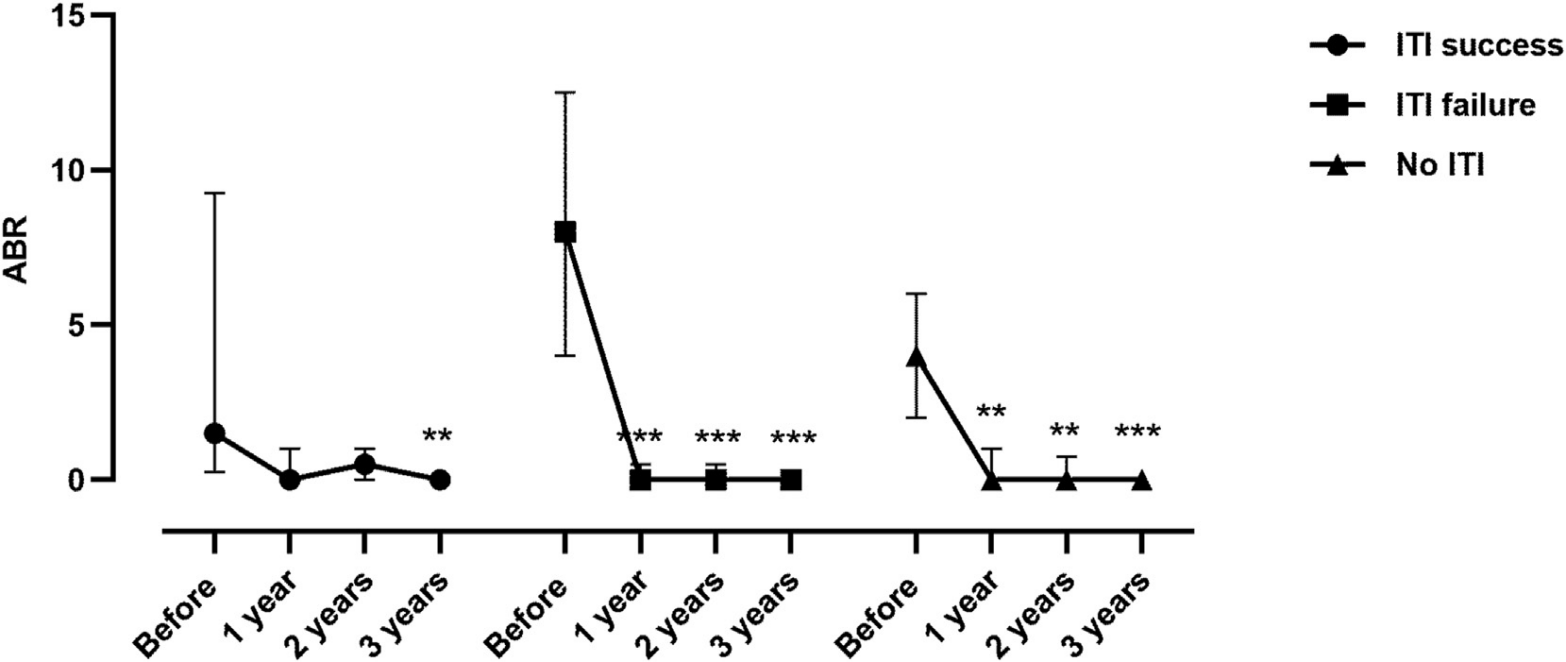
The data are presented as median and IQR. Within each group, comparisons vs “before” were performed by Friedman test followed by Dunn’s post hoc test. ∗∗*P* < .01, ∗∗∗*P* < .001. ABR, annual bleeding rate; ITI, immune tolerance induction.

The median time on emicizumab prophylaxis was 3.3 years (range, 2-4 years), similar among all patient groups, as shown in Table 2. Notably, most patients initiated emicizumab a long time after ITI completion (Table 1). No difference was observed with regard to annual bleeding rate after initiation of emicizumab prophylaxis across participant subgroups (Figure 1).

**Table 2:** Clinical outcomes of our patients.

Clinical outcome	Failed ITI *N* = 15	Successful ITI *N* = 15	No ITI *N* = 21	*P* value
Time on emicizumab, median (IQR) (y)	3.8 (3.3-4.1)	3.0 (2.3-3.2)	3.1 (1.3-3.5)	<.001
ABR after 1 y after Emicizumab initiation; median (IQR)^b^	0 (0; 0.25)	0 (0; 1)	0 (0; 1)	.99
Inhibitor after 3 y median (IQR)	2.0 (1.1-12.0)	0.0 (0.0-0.5)	0.6 (0.0-5.0)	<.001
No. of patients exposed to FVIII^a^	4 patients, 4 bleeding episodes	8 patients, 8 bleeding episodes	8 patients, 9 bleeding episodes	
No. of patients exposed to FVIIa^a^	1 patient, 4 bleeding episodes		1 patient, 6 bleeding episodes	

ABR, annual bleeding rate; F, factor.

a FVIII or FVIIa exposures (1-2 exposure day(s) per event) were related to treatment of bleeding episodes.

b ABR was calculated on the basis of the number of documented treated bleeding events per year.

During the study period, 22 of 51 participants experienced 31 breakthrough bleeding episodes. Patients usually received 1 to 3 doses (median, 1 ED) of any coagulation concentrate per bleeding event.

Four participants with low responding FVIII inhibitor (<2 BU) underwent minor surgical interventions, being exposed to 1 to 2 doses of FVIII. No increase in FVIII inhibitor levels was noted following any of these procedures.

**Figure 2:**
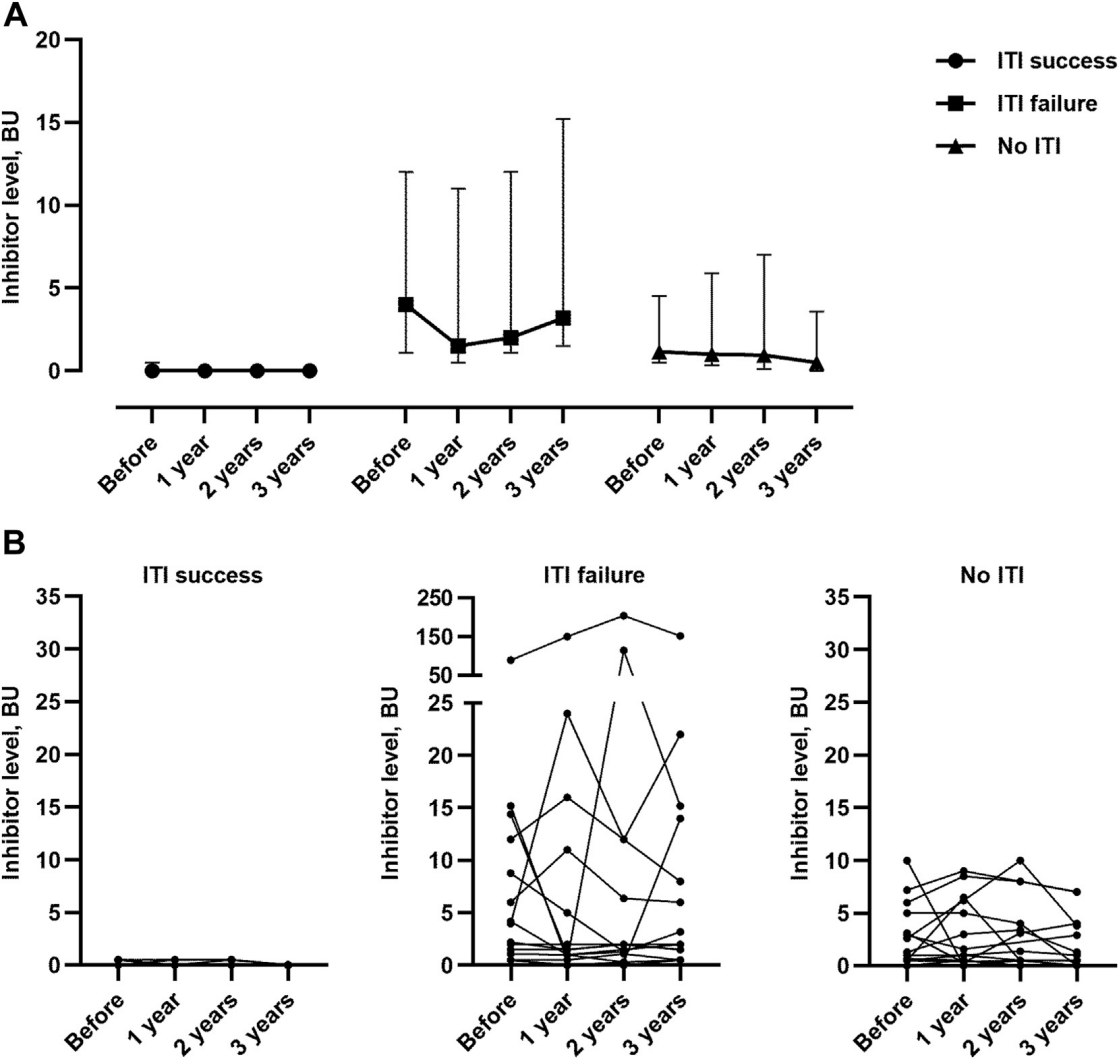
(A) Inhibitor levels in different cohort subgroups before and during emicizumab prophylaxis. The data are presented as median and IQR. Within each group, comparisons vs “before” were performed by Friedman test followed by Dunn’s post hoc test. No statistically significant difference was detected. (B) Inhibitor levels before and during emicizumab prophylaxis. In these graphs, each line represents an individual patient. Inhibitor levels were measured as BU/mL. “Before” refers to inhibitor levels prior to emicizumab initiation. ITI, immune tolerance induction.

Figure 2A demonstrates median inhibitor levels in the different subgroups before and during emicizumab prophylaxis. Figure 2B presents the individual changes in FVIII inhibitor levels in each of the participants.

Among the participants who failed ITI (*n* = 15), there were 5 participants whose inhibitor levels increased during the study period. One of these participants had a motor vehicle accident due to which he was exposed to 8 doses of FVIII infused daily for 7 days to treat pelvic fractures and abdominal hemorrhage. Emicizumab level at the time of the accident was 28 mg/dL, and trough FVIII levels were kept around 50%. In this patient, the inhibitor level increased from 5 to 15 BU following FVIII exposure for 8 EDs (noted after FVIII was stopped). Three other participants exhibited increased inhibitor levels following FVIII exposure for 1 to 2 ED(s) due to traumatic bleeds. In these participants, FVIII inhibitors were transient and returned, within 6 months, to their baseline inhibitor levels. In 1 participant, the increase in inhibitor level from 0.5 to 2 BU occurred without any exposure to FVIII, followed by spontaneous decrease to 1 BU.

In the “No ITI” group, 8 of 21 participants were exposed to FVIII (1-2 ED(s)) in order to treat breakthrough bleeds.

In general, FVIII inhibitor titer mostly decreased and fell into the low titer range in “No ITI” patients and in some “failed ITI” participants. Among participants exposed to FVIII, transient anamnestic responses were noted, with inhibitor decreasing to baseline after about 6 months—see Figure 2. In 1 participant from the “No ITI” group who received FVIII following a head injury, the inhibitor titer increased from 2 to 7 BU. Following this event, he received recombinant FVIIa instead of FVIII for treatment of another traumatic bleed. Nonetheless, his inhibitor level eventually decreased due to no further FVIII exposure. None of the other participants had to switch to bypass agents’ therapy during the study period.

Another participant was not exposed to FVIII, yet his inhibitor level increased from 4 to 12 BU and decreased back to baseline within 3 months. In all the other participants, the inhibitor level remained relatively stable.

In the group of participants who underwent successful ITI, 8 of 15 participants were exposed to a total of 13 EDs (1-2 ED(s) each) for traumatic breakthrough bleeds. In all these participants, the inhibitor levels remained zero, indicating successful tolerance maintenance despite exposure to FVIII.

Overall, after 3 years of follow-up, inhibitor levels declined in all participant subgroups. In general, exposure to FVIII did not affect the inhibitor levels. Notably, during the first year of emicizumab prophylaxis, 13 participants were exposed to FVIII. Their inhibitor levels (median [IQR] vs 38 participants who were not exposed: 0.5 [0-1.75] vs 0.5 [0-4.85]) were similar (*P* = .10). Nonsignificant differences in FVIII inhibitor levels following FVIII exposure (*n* = 20 in the second year and *n* = 22 in the third year) compared with participants without FVIII exposure (*n* = 28 and *n* = 23 in the second and third year of follow-up, respectively) were observed over longer follow-up of emicizumab-treated participants.

## 4 Discussion

Our longitudinal follow-up of patients with HA shows that patients who underwent successful ITI retained tolerance, with no documented inhibitor recurrence following occasional exposure to FVIII. However, partially tolerized patients may experience transient anamnestic responses.

Notably, current international and national guidelines still recommend an ITI attempt in patients with SHA who develop inhibitors [8,19,20]. A recent European survey indicated that ITI is still the mainstay of treatment for patients with HA and inhibitors. Emicizumab prophylaxis can be considered a cost-saving treatment for patients with HA and inhibitors during ITI [[21], [22], [23]].

Using emicizumab in lieu of ITI has been well described in pediatric patients [20,[24], [25], [26]]. The “Atlanta protocol” suggested that ITI can be safely administered in pediatric patients with HA and inhibitors treated with emicizumab, yielding a clinical decline in inhibitor titer over time [27]. Further pediatric and adult cohorts of patients with inhibitors followed up for longer periods confirmed the aforementioned conclusions [26,[28], [29], [30]]. The decline in FVIII inhibitors over time, noted in our patients as well, may stem from lack of FVIII exposure.

There is still a lack of data about treatment strategies after inhibitor development as well as safety and efficacy of lower FVIII dosing/frequency regimens combined with emicizumab prophylaxis. Thus, decisions regarding initiation and administration of ITI, especially in newly diagnosed patients, have become much more complicated [30,31]. The planned MOTIVATE study aims to capture the efficacy and safety of 3 different approaches for managing patients with HA and inhibitors: ITI without emicizumab, ITI in combination with emicizumab, and emicizumab alone with no ITI attempt [32].

Interestingly, all the parents of children with newly diagnosed FVIII inhibitors followed up at our center chose to defer ITI and eventually avoid it, favoring emicizumab prophylaxis alone. Patients who did not receive ITI were significantly younger at the time of emicizumab prophylaxis initiation than those in the subgroup of previously attempted ITI. This highlights the change in management at our center, which is driven by the availability of good prophylactic nonreplacement therapies.

Giving up ITI treatment in the era of emicizumab is a debatable topic that deserves further attention.

Currently, the best approach to maintain tolerance after successful ITI remains to be elucidated. Whether regular FVIII exposure should still be required is yet to be determined. Notably, in all patients after successful ITI, receiving emicizumab prophylaxis, and being followed up for over 3 years, tolerance has been preserved with no inhibitor recurrence despite repeated short-term FVIII exposures. There was no difference in inhibitor levels among patients with brief FVIII exposure and no FVIII exposure, as demonstrated by repeated examinations during the entire study period.

Data regarding potential inhibitor recurrence in emicizumab-treated patients after ITI are somewhat conflicting. Sporadic inhibitor relapses have been reported by Doshi et al. [33] and Hassan et al. [26] among patients with previously successful ITI who transitioned to emicizumab prophylaxis. In contrast to these reports, in 11 of 12 tolerized patients, inhibitor recurrence was reported in 1 partially tolerized patient only after FVIII exposure, whereas among 5 of 6 patients re-exposed to FVIII for bleeds or surgeries, tolerance was preserved. The duration of follow-up was 14.2 ± 6.1 months only with 1 to 30 ED(s) to FVIII [24]. These results are similar to our findings. This conundrum may be solved by the PRIORITY study, which aims to assess inhibitor recurrence after 2 years of nonexposure vs continued FVIII exposure along with emicizumab prophylaxis in children followed up after successful ITI [34].

As expected, we observed elevations of FVIII inhibitor titers after FVIII exposures among our nontolerized patients. However, it was encouraging to realize that FVIII inhibitor levels had subsequently decreased with further lack of FVIII exposure.

Interestingly, we saw 3 patients in whom FVIII inhibitor levels were transiently increased without any obvious reason and without any FVIII exposure (similar to Doshi et al. [33]). The demographic or clinical profile of these patients did not differ from the rest of the cohort, yet their immunologic background deserves further attention and should be further investigated.

Despite the fact that FVIII inhibitor recurrence is not expected to occur in the vast majority of tolerized patients, ongoing inhibitor monitoring in patients post-FVIII inhibitor who transition to emicizumab therapy is currently strongly recommended [27]. Our study supports the notion that patients with HA and a history of successful ITI may be treated with emicizumab prophylaxis alone without additional FVIII exposure. As inhibitor recurrence was not noted among such patients following short-term “danger signals” leading to an on-demand FVIII exposure, we suggest that management of breakthrough bleeds and minor surgical procedures may be safely performed without affecting patients’ tolerance. On the other hand, patients who were never fully tolerized may experience re-emergence of FVIII inhibitors following FVIII exposures. Therefore, inhibitor recurrence monitoring in the surgical setting is crucial for such patients as results are expected to guide proper hemostatic therapy such as use of bypassing agents. The difference between patients who were fully tolerized and those who never completed successful ITI suggests that there is still a role to play for ITI among patients with newly diagnosed FVIII inhibitors. Nonetheless, ITI could be deferred in young children or special patient populations, with emicizumab serving as bridging therapy.

This study had limitations. First, as our patients were monitored for a median of 3 years, our results have to be further followed up. Second, the majority of fully tolerized patients initiated emicizumab prophylaxis after a median of 13 years after successful ITI; therefore, our study results may not be applicable to recently tolerized individuals. Last but not least, most of our patients experienced 1 to 2 ED(s) only; thus, our results should not be extrapolated into the surgical setting or scenarios of severe bleeds wherein prolonged FVIII exposure may produce persistent “danger signals” [35].

Our study results suggest that following successful ITI, FVIII prophylaxis perhaps may not be required for tolerance preservation in emicizumab-treated patients. Further data from future systematic clinical trials and postmarketing studies are required to guide sound future practice [19,32,[36], [37], [38], [39]].
