# Supplementary material for: Factor VIII inhibitors in hemophilia A treated with emicizumab: longitudinal follow-up of outcomes
Source: Res Pract Thromb Haemost. 2023 Jun 14;7(4):100278. doi: 10.1016/j.rpth.2023.100278 (PMC10394563; doi:10.1016/j.rpth.2023.100278)
Supplement: Supplement table 1 [file mmc1.docx]

**Supplement table 1**

Supplement demographics and clinical data of emicizumab treated patients

| **No ITI**  **N=21** | **Successful ITI**  **N=15** | **Failed ITI**  **N=15** |  |
| --- | --- | --- | --- |
| - Inversion22 – 10 pt. - c75C>G p.Tyr25* - 1 pt. - c.6976C>T p.Arg2326 – 1 pt. - deletion of exon 1-14 – 1 pt.   unknown – 9 pt. | - Inversion22 – 10 pt.   unknown – 6 pt. | - Inversion22 – 9 pt. - c.3439C>T p.Gln1147*(Q1147>stop) - 1 pt. - deletion of exon 8+9 – 1 pt. - deletion of exon 14-21 – 1 pt.   unknown – 2 pt. | **Mutations** |
| - Two teeth extraction ( 1 BU, 0.6 BU) |  | - Port extraction ( 2BU) - Close fracture reduction (1BU) | **Surgeries during emicizumab therapy in patients with inhibitors #** |

# In this table we refer to low responding inhibitor patients who were exposed to FVIII concentrates peri-operatively

*Detailed patients' demographics appear in table 1

ITI- immune tolerance induction
